# Barriers to, and enablers of, participation in the Allied Health Rural and Remote Training and Support (AHRRTS) program for rural and remote allied health workers: a qualitative descriptive study

**DOI:** 10.1186/1472-6920-14-194

**Published:** 2014-09-19

**Authors:** Wendy H Ducat, Vanessa Burge, Saravana Kumar

**Affiliations:** Cunningham Centre, Division of Allied Health, Darling Downs Hospital and Health Service, Toowoomba, Australia; International Centre for Allied Health Evidence, School of Health Sciences, University of South Australia, Adelaide, Australia

## Abstract

**Background:**

Allied health professionals play a critical role in enhancing health outcomes in primary and tertiary settings. Issues affecting the recruitment and retention of allied health workers in rural and remote areas are multifactorial. Access to relevant and effective continuing professional development is argued to be a recruitment and retention strategy for health professionals in non-metropolitan areas, however trial of the effectiveness of professional development programs and identification of enablers and barriers to participation is needed. The Allied Health Rural and Remote Training and Support (AHRRTS) program aimed to provide an integrated program of education and professional support activities for allied health professionals working within Queensland Health in rural and remote locations. The aim of this study was to explore enablers and barriers to access to, and effective participation in, the AHRRTS program from various allied heath stakeholders’ perspectives.

**Methods:**

A qualitative descriptive study utilising semi-structured interviews with various allied heath stakeholders was undertaken. The interview questions focussed on a number of issues pertinent to AHRRTS program, with specific probing questions regarding barriers and enablers to participation in the AHRRTS program. The interviews were then transcribed verbatim and analysed using thematic analysis.

**Results:**

Using purposive sampling, a total of 55 stakeholders were interviewed for this study. Time, organisational factors and travel were identified as common barriers and organisational factors, travel and presentation modes were identified as common enablers. Interestingly, some of these factors act as barriers and/or enablers highlighting that these are essentially two sides of the same coin. The findings suggest that while it is important to have policies and procedures for ongoing support of allied health professionals, it should be complemented by enabling strategies to address persistent barriers.

**Conclusion:**

The study suggests that while a program such as AHRRTS is accessible and facilitates participation in a number of ways, significant barriers to participation continue to persist at the coal face. Addressing these barriers will require a targeted, multifaceted approach. Lessons from this study provide unique insights into factors which influence the successful implementation and sustainability of recruitment and retention initiatives for rural and remote allied health workers.

## Background

Health care in Australia faces a number of unique challenges. Armstrong et al. [1] summarise eight key challenges including a universal recognition of the need to focus on chronic disease prevention and management and demonstrable workforce challenges in regional, rural and remote areas of Australia. These authors state “shortages are more significant in outer metropolitan, rural and remote regions, especially in Indigenous communities, and in particular areas of care such as mental health, aged care and disability” (p.486). The inequity in the health status of Australians who live in rural and remote Australia is of national concern, with rural and remote Australians having poorer health status, less access to health professionals [2] and less access to private health services [1] than their metropolitan counterparts.

Allied health professionals play a critical role in enhancing health outcomes in primary and tertiary health settings, providing independent and multi-professional services in chronic disease management, rehabilitation, palliative care, mental health, health promotion and care planning [3]. Despite their important contribution, problems with access to effective allied health services in rural and remote areas persist. While the ideal staffing ratio for allied health has not been well researched and lags behind literature for medicine and nursing [4], available research shows that the allied health workforce is significantly underrepresented in rural and remote areas of Australia based on a practitioner to population ratio method [5].

Despite concerted efforts, there are ongoing issues in the recruitment and retention of this diverse workforce. Some of the barriers to recruitment include social, professional and geographical isolation, lack of resources, high workload, and complexity of caseload and workplace stress for rural and remote clinicians [6–8] as well as requisite personal attributes and skills to effectively working in a rural and remote area including flexibility, collaboration and cultural awareness [9]. Organisational efforts to recruit and retain rural and remote allied health practitioners by addressing amenable areas for change are needed [2, 3, 5].

While issues with recruitment and retention in rural and remote areas are multifactorial, some factors are more amendable to change than others, for example, access to education, training and professional support within the organisation [10]. Allied health professionals in rural areas of Australia are dissatisfied with access to appropriate continuing professional development opportunities and professional support and turnover intention is high [6, 11, 12]. Lack of training and support, difficulties with local access and/or prohibitive costs associated with offsite training attendance, inadequate discipline specific support and a lack of backfill opportunities are some of the issues facing non-metropolitan health practitioners [3]. Access to relevant and effective continuing professional development is argued to be a retention strategy and an incentive for future health practitioners to seek employment in non-metropolitan areas; as well as a quality mechanism to ensure a capable and confident workforce [2, 3], however trials of the effectiveness of professional development programs and their associated enablers and barriers to participation is needed.

The Allied Health Rural and Remote Training and Support (AHRRTS) program coordinated by the Cunningham Centre, Division of Allied Health, Darling Downs Hospital and Health Service was designed to provide coordinated, capability based education, training and professional support to the rural and remote allied health workforce in Queensland Health. Eligibility for the AHRRTS program was dependent on the allied health professional’s primary work location. The program was available to allied health professionals in all categories of employment (full-time, part-time, casual, permanent and temporary).

One of the drivers for the development and implementation of the AHRRTS program was the poor uptake and variability in the access to existing programs for rural and remote allied health practitioners and the lack of a coordinated program of capability development and skill maintenance for the rural and remote allied health workforce. The intention of the AHRRTS initiative was to provide a holistic program of clinical education, training and professional support strategies specifically focused on the development and maintenance of capabilities required for rural and remote allied health practice.

The AHRRTS program design incorporated existing as well as new initiatives to form an integrated framework for education and support for rural and remote allied health. Components of the AHRRTS program include distance-based (e.g. videoconference, teleconference, online) and face-to-face delivery of training based on individual practitioner and/or team requirements. AHRRTS offerings cover eight domains of capability training, namely service delivery, equity and diversity, professional skills, ethical practice, development and support, quality and safety, clinical management and clinical skills in line with the Allied Health Rural and Remote Allied Health Capability Framework [13]. Program participation is flexible and tailored to the specific requirements of each rural and remote allied health professional who enrols. The AHRRTS program also coordinates professional support activities including professional supervision and mentoring matching as well as funding for face-to-face supervision meetings and incentives for rural or remote supervisors who are providing support outside their hospital and health service. In addition, organisation and funding for professional clinical placements to up-skill rural or remote clinicians in service areas relevant to their local service delivery is coordinated through the sister program, the Allied Health Professional Enhancement Program (AHPEP).

The AHRRTS program was formally launched in Queensland Health in February 2010 and over the course of the initial two-year trial, over 170 out of 380 eligible allied health professionals participated in the AHRRTS program. A formal evaluation of the AHRRTS program was conducted by an independent organisation which investigated the impact and uptake of the AHRRTS program. An important component of the evaluation was the exploration of enablers and barriers to access to, and effective participation in, the AHRRTS program from various allied health stakeholders’ perspectives and this manuscript provides an overview of these findings.

## Methods

A qualitative descriptive research design [14] was used to explore participants’ perspectives of barriers and enablers for their participation in the AHRRTS program. As we wanted to capture participants’ experiences in their own words using their own language and descriptions, this was an ideal design of choice.

### Ethics

Ethical approval for the statewide evaluation was obtained from the Human Research and Ethics Committee of the Prince Charles Hospital, Queensland Health, and the Human Research Ethics Committee of the University of South Australia.

### Participant selection

Potential participants were key stakeholders within Queensland Health and included allied health professionals, their managers, supervisors and mentors. Participants were purposively identified through the AHRRTS program and were invited to participate in this research project. Purposive sampling was chosen in order to ensure diversity of participants, and that participants represented a range of allied health disciplines. Using this framework, a sample of 55 stakeholders including nine line managers/directors, four professional supervisors/mentors and 42 rural and remote allied health professionals who had been involved in the program for at least six-months participated in this research. The disciplines represented by the 42 AHRRTS program participants are displayed in Table 1.

**Table 1:** **Allied health disciplines of AHRRTS program participants in the study**

Allied health discipline	Number of study participants
Dietetics	4
Medical radiation professions	2
Nutrition	7
Occupational therapy	8
Physiotherapy	6
Psychology	1
Social work	11
Speech pathology	3

### Data collection

Following informed consent, semi-structured interviews were conducted with participants at their nominated time and place. The semi-structured interviews were conducted over the phone by a member of the research team trained in a standardised semi-structured interview protocol. The interview questions focussed on a number of issues pertinent to AHRRTS program, with specific probing questions regarding barriers and enablers to participation in the AHRRTS program. Each interview took between 20–40 minutes and was recorded. The interviews were then transcribed verbatim by an independent transcribing service. The transcripts were de-identified in order to ensure participant confidentiality.

### Data analysis

Thematic analysis [15] was undertaken as the primary means of data categorisation for the purposes of classification, summarisation and tabulation. The analysis of data was interpretive, that is, the data was investigated for what was actually meant as the response to the question, what was inferred and how it was implied. Thematic analysis involved transcripts being read repeatedly with preliminary coding to identify emerging concepts and ideas with similar ideas being grouped together to form codes. Following systematic process of coding, similar codes were then connected to form categories and common categories were identified and grouped to form themes [16]. The process of thematic analysis involved continually revisiting the data and reviewing the categorisation of data until the researcher was sure that the themes and categories used to summarise and describe the findings were a truthful and accurate reflection of the data. A qualitative data management system, Nvivo9 software™ [17], was utilised in the data storage and analysis process.

In qualitative research, as part of ensuring rigour in its research methods, trustworthiness is an important concept to consider. Trustworthiness relates to a number of different constructs including credibility (the level of confidence in the truth of the research findings), transferability (the applicability of the findings to other contexts), dependability (the consistency and repeatability of results from the research) and the influence of the researcher on the result of the research [18].

Recognising the importance of trustworthiness in qualitative research data collection and analysis, a number of key processes were put in place for this evaluation research. These processes have been widely recommended within the qualitative research literature as means of ensuring rigour [18–20]. The strategies employed in this research project were:

- ● adherence to a standardised data collection protocol including the semi-structured interview protocol used during the interview process
- ● all interviews were audio taped and transcribed verbatim by an independent typist with subsequent validation by the interviewer
- ● all interviews were monitored for consistency of data collection methods including either an independent observer regularly monitoring the process by sitting in on interviews or a second review of the audio recording to ensure adherence to the interview protocol during interviews
- ● coding of the qualitative data was undertaken by more than one coder and consistency between coders was ensured (cross checking). If there were differences between the codes, these were discussed until consensus was reached
- ● thick descriptions in the form of meaningful quotations to represent important themes and categories were included when reporting data analysis

## Results

### Enablers and barriers to effective participation in AHRRTS

Thematic analysis of the qualitative data indicated key themes pertinent to enablers and barriers to access to, and effective participation in, the AHRRTS program. *Identified barriers* related to factors which negatively influenced access to and effective participation in the AHRRTS program for allied health professionals. *Identified enablers* related to factors which positively influenced access to and effective participation in the AHRRTS program for allied health professionals. Within barriers, three key themes were identified. They were *time, organisational factors and travel*. Within the enablers, three key themes were identified. They were *organisational factors, travel and presentation mode.*

### Time as a barrier

Time was an important barrier commonly reported by allied health professionals, line managers and supervisors. Informants’ views were that limited time was a significant barrier in accessing and participating in the AHRRTS program. Many informants commented that there were competing priorities for their time (clinical vs. non clinical) and often this resulted in them not being able to participate, or discerningly choosing what to participate in, within the AHRRTS program. An allied health professional commented that,

*“Time is a really big one for me especially too, before the Senior Physio was here and because we don’t have an admin staff or anything, being the sole Physio, you’ve got your clinical load as well as the administration load and I also outreach”*

Another allied health professional commented on prioritising his/her time to match clinical load.

*“Again it’s just that clinical time. And for me, clinical work will always, depending on what it is, and the urgency, will always take precedence over going and attending something.”*

Line managers also seemed to echo this view that in many instances clinical issues and time spent with patients tend to override time for personal development.

*“I’ve got a lot of competing interests and I have to really pick and choose what I do so when it comes to the AHRRTS program.”*

Many informants also commented that existing staffing levels meant that many were not in a position to participate in the AHRRTS program due to their status as sole practitioners and lack of availability for backfill.

*“Well, we don’t really go anywhere long enough - we avoid going anywhere long enough that would require backfill. So we don’t go to that much that it affects us. It does have an effect on our workplace afterwards, catching up.”*

Allied health professionals also commented about being sole practitioners and its effect on their participation in the AHRRTS program. The issue of competing priorities for a sole practitioner was highlighted by a line manager,

*“The problem with me is that working as a sole physio up here, being responsible for over 3500 people but only having 24 hours; it’s hard for me to take the time away from all my other responsibilities and sit in front of a video conference.”*

Difficulties associated with staffing levels were also highlighted as a barrier to accessing AHRRTS program in rural and remote areas. Having a small number of staff, who had to manage their time between treating patients and participating in professional development activities were seen as a constant challenge. An allied health professional commented that,

*“I suppose time also affects my ability to participate in those support programs because being only .5 you need to get out to those towns and so sometimes you cannot participate in education sessions or professional support because you need to get to those town and see clients.”*

A line manager commented that,

*“When you’re only working .6 it’s very hard to find time within your schedule. Like it takes an hour every week to do your supervision and then if you have a video conference it’s another 30 minutes or another 60 minutes. Plus there are a lot of other demands. I’ve got video conferences coming from all directions.”*

### Organisational barriers

The second theme identified from the interviews was *organisational barriers* to access to and effective participation in the AHRRTS program. Organisational barriers reflected factors within the organisation which hindered effective participation in AHRRTS programs. A common finding was the difficulty associated with accessing appropriate videoconferencing facilities. One line manager commented that,

*“With the video conferences they, they’re generally really good, biggest issue is accessing a video conference machine.”*

More commonly, though, many informants highlighted poor managerial support for participation in the AHRRTS program. Informants commented that they had to convince their managers that professional development was a worthwhile exercise and get them to “sign off” on these activities. One allied health professional commented that,

*“That’s the main thing that I struggle with across the board is just that being able to just go and do it and not be drilled about why you’re doing that because it’s our district team, but why are you doing it.”*

A line manager also echoed similar views with particular reference to new graduates. They commented on the need to support these new allied health professionals and at times this was not forthcoming from his/her organisation.

*“For line managers just so the willingness to let people go, particularly new graduates to give them time to because there’s a lot of training options available and when they start seeing the days add up they start questioning and it just needs the support from top down and that was probably one of my hurdles trying to get the staff off to some of the things.”*

Administrative and budget difficulties were also highlighted as an important barrier within the organisational theme. One allied health professional commented that despite support from line managers, ultimately availability of funds dictated this.

*“Basically just budgets. All my managers are really supportive of any sort of PD, professional support that we can do but it basically comes down to the budget.”*

### Travel as a barrier

The third barrier identified to access to and effective participation in the AHRRTS program was travel. This theme related to issues associated with travelling for professional development which hindered participation for many allied health professionals.

One allied health professional recounted his/her first experience of attending an AHRRTS program. They said that,

*“I only just last week went to my first professional development workshop which was a two day workshop in Brisbane on Thursday/Friday…….would’ve like to have gone to more just day workshops and that, but really it is the travel and the distance that I haven't.”*

This view was also supported by a line manager who commented on the increasing costs that were associated with travelling for professional development activities and how they had to prioritise participation in these activities.

*“If there’s a workshop say in [metropolitan city], well in the current climate no one will be able to attend, they’ll have to do it on their own time. If it’s in “regional city”, I’ll probably be able to send one person. If it’s held locally I can send a whole team because it’s only the day’s work that’s affected, whereas to go to [regional city] you lose two days and you’ve got accommodation and travel and transport and those sorts of things.”*

Informants discussed a number of enabling factors which could assist in promoting access to and effective participation in the AHRRTS program. Within enablers, three key themes were identified. Theme one related to *organisational enablers*, or factors within an organisation which may assist in the participation and uptake of activities supported by AHRRTS program. Theme two related to *travel*, or the enabling factors associated with travelling. Theme three related to *mode of presentation* which enabled access to and participation in the AHRRTS program activities.

### Organisational enablers

Within organisational enablers, a theme that was most commonly reported by many informants was the importance of support from their line managers. Many commented that if the line manager was supportive of participation in the AHRRTS program, this was likely to occur. Alternatively, if this was not the case, participation was likely to be hindered. One line manager commented that,

*“I think if your line manager and our Director of Allied Health in our district really value or can see the benefit of AHRRTS so it’s well supported in our district for people to have that protected time to link into the videoconferences too. …….if you didn’t have supportive line management then it might be a challenge for some people because they’re not given that time off line but our team certainly is.”*

This was also reflected by allied health professionals who valued the support provided by their line managers and team leaders who supported them to participate in conferences and training workshops.

*“So I have an amazing line manager and our director is awesome as well. So between the two of them, if I feel the training is valuable, I get to go.”*

Support from the state department of health was also identified as an important organisational enabler in participating in AHRRTS programs. Participants commented that given that the AHRRTS program was endorsed by the state health department and was intrinsic to this state, this facilitated their participation in training activities.

*“The fact that it (AHRRTS program) is internal, I suppose makes it a little bit more, you know you’ve got that support to go yes, you should do this because it is endorsed by Queensland Health. I think if you were an external program, it might influence things a bit differently, might change things.”*

Many participants commented that funding support provided by the AHRRTS program meant there was little financial burden for local health services as part of allied health professionals participating in the AHRRTS program. An allied health professional commented that,

*“I think financially, because the district didn’t have a problem with me going either because it was covered financially, so it wasn’t coming out of the district budget. So, I was allowed to go.”*

Similar views were also echoed by line managers who highlighted that having another organisation contribute to the professional development of their staff facilitated its uptake.

*“Having another organisation or fund an organisation to provide these services, whether it be whole of government or somewhere, it takes off that day to day worry about coming in under budget and how do you get everything done, so that was a big, that was great, somebody’s taking on the or being the champion for the allied health team.”*

Supervisors were also positive for the funding support provided by the AHRRTS program to promote participation in supervision and mentoring activities. A supervisor, whose supervisee was geographically distant from her, commented about these funding opportunities.

“I recently applied for some funding to be able to go out and spend a couple of days with ***** in [rural location] and knowing that someone was willing to pay for those costs was really great, unfortunately I had to cancel that visit due to some things at ***** end. But it was good to know that the programme would support that.”

Many participants also commented positively about the AHRRTS program driving professional development for rural and remote allied health practitioners and its benefits in promoting participation and uptake. One of the line managers commented that, given his/her non-allied health professional background, having AHRRTS take the ownership for professional development of his/her allied health staff was a significant enabler. He comments,

*“I think it was a great initiative. I’m a nurse ………so when it came to the professional development side in my allied health team I didn’t have a lot of knowledge in that area and along came the AHRRTS program and with the leader of the allied health team for the district, solved a lot of my worries and was able to help facilitate that.”*

Another line manager also commented on the efficiencies involved in having AHRRTS programs being centrally coordinated and delivered. The time saved can then be put to use in other areas more efficiently (such as client services). They commented that,

*“At other rural and remote places I’ve worked we have initiated our own education because our needs are so specific but that took a lot of time and then we had to write up the proceedings and it was often done in our own time. So this just makes things a lot more efficient which means that we can do client services.”*

### Travel as an enabler

Theme two within the enablers was travel. allied health professionals commented that being able to travel away from their workplace meant there was dedicated, undisturbed time for professional development and opportunities for networking. This was an important enabler as allied health professionals saw this as an opportunity to learn, reflect and bond with fellow team members.

*“The best training that I find is to really get away from the workplace to actually do it. That’s probably the biggest factor. Because if you’re not there at the work, if there’s a crisis that happens someone else from the team has to deal with it and you don’t have the interfering phone calls like a client might be phoning up saying ‘can you do this for me’ or helping them in some area.”*

Another allied health professional commented on the bonding opportunities travel away from work provided. They commented that

*“so it was really nice to be able to travel as a team and we got to know each other quite well and we all bonded as a team quite well from that Rural Skills Conference because we had to travel together, yes.”*

### Presentation mode as an enabler

Theme three within the enablers was the mode of presentation. Having access to videoconferencing was an important enabler, which overcame numerous barriers (associated with travel, cost, staffing). Many participants reflected on the opportunities presented by videoconferencing and the flexibility it afforded them.

*“I think that the delivery by video conference was exceptionally handy. I’ve spent so much time away from my base at face-to-face training and professional development, it’s good to be able to just duck down to the VC machine for a couple of hours and still be able to do close to a full day in the office and not have to take that travel time.”*

Line managers too agreed with such statements as having access to videoconferencing provided opportunities which otherwise may not be accessible and also meeting the requirements of their allied health staff.

*“The use of videoconferencing and teleconferencing is just, it’s vital and it really underpins a lot of what AHRRTS does so I think that’s been great and a lot of the programs under AHRRTS are very flexible so whether it be mentoring or videoconference series and those sorts of things have been very flexible that where people can link into them in a variety of ways so that’s been brilliant.”*

## Discussion

An accessible, confident and capable rural and remote allied health workforce is necessary to meet the current key challenges of health care provision in Australia. Previous research has highlighted the need for organisational initiatives to ameliorate professional isolation for rural and remote health providers and increase coordination of training and development to support best practice [6–8]. These studies have called for coordinated programs that are tailored to meet the unique requirements of rural and remote health services and their patients.

This study reveals important barriers and enablers to successful engagement in a rural and remote specific professional development program tailored to support the rural and remote allied health workforce in Queensland, Australia. One factor for successfully engaging in AHRRTS was organisational support including the general perception of support from the broader public service organisation, as well as work-unit specific support from local line managers, and AHRRTS program specific support including coordination, funding, and accessible training offerings. These findings are in agreement with previous evidence that broad organisational initiatives such as professional development allowance and leave; policies and guidelines including performance appraisal and development processes are important aspects of employment contracts [6].

The AHRRTS program’s focus on continuing professional development tailored to the learning requirements of individual health practitioners across the span of their careers provided a structured pathway to engaging in professional development for informants. Informants indicated that AHRRTS program coordination or “ownership” for supporting their professional development was a key enabler to their participation. This highlights the importance of a centralised agency coordinating and delivering such programs. These in addition to key funding provisions within the AHRRTS program, including for face-to-face supervision meetings and some travel bursaries for rural skills training, were seen as enablers.

It is interesting to note the emphasis placed by informants on the support from local line managers as a key enabler and this is consistent with previous research [21]. Good relationships with line managers, approvals to attend professional development, clarity around expectations and line managers who engaged in organisational policies listed above were all key factors to enable successful participation in the AHRRTS program, particularly in certain regions over others. Travel was a theme that was cited as both an enabler and a barrier. Informants reported that travel to attend professional development was an important enabler as it allowed demarcation and prioritisation of the professional development from other workplace demands for the specific period of time.

The final enabler that emerged was presentation mode and it was clear from the evaluation that videoconferencing and distance based forms of delivery are critical for the rural and remote workforce to allow effective access to professional development. With the increasing role of technology and distance based training, it is not surprising that such modes of presentations were valued by the informants.

However, it does raise an interesting paradox. While travelling away (thereby being physically removed from their workplace) was considered by some of the informants as an enabler, use of videoconferencing and distance based forms of delivery (thereby physically present at their workplace) were considered by others as an enabler. This dichotomy highlights the need for a flexible approach of such programs, which caters for a wide range of learning styles – as well as other factors that prohibit travel, for example, family commitments - and the perils of a “one-size fits all” approach.

Time was the most often cited barrier to participation, though the underlying analysis of this construct showed that participants had diverse meanings attributed to “not having enough time” or “needing more time”. Both internal and external pressures impacted on participants’ “lack of time”. Internal sources included pressure on self to justify time spent on professional development (guilt, perfectionism, altruism) and lack of knowledge regarding the “right” balance of direct client care and professional development. A theme expressed by many informants was problems managing competing clinical versus non clinical priorities.

Prioritisation of time was clearly an issue with multiple tasks such as direct client contact, outreach travel and administration duties conflicting with allowing time for professional development and supervision. These findings are supported by previous research by Dawson et al. [22] and Hyrkäs et al. [23]. A practical implication of this theme is that “not having enough time” is multifaceted and organisational initiatives should be tailored to meet the diverse challenges. Informants also acknowledged organisational issues and travel as barriers to access to, and effective participation in, the AHRRTS program.

Organisational issues continue to pose significant challenges for informants and there is research evidence which indicates the impact of organisational culture on support activities such as clinical supervision [24]. It is not merely sufficient to create professional development programs and opportunities without creating an organisational culture which supports and endorses engagement and participation.

### Limitations

This research reported on the perspectives of various stakeholders, who represented a range of allied health and other health disciplines, as a whole. While this could be considered a strength (rich diversity among informants), it may also be viewed as a weakness, as it did not provide specific information about individual allied heath disciplines. Some of the interviews were conducted by project officers of the AHRRTS program and therefore this may have inhibited informants from fully disclosing their perspectives of the AHRRTS program. This is not a significant limitation though as the data collected by the project officers of the AHRRTS program did not differ from those collected by independent researchers.

## Conclusions

There is increasing international recognition that participation in continuing development initiatives such as the AHRRTS program are important to enhance safe and evidence based practice [25], particularly in non-metropolitan health settings that have less resources and high complex demands [26]. In conclusion, this paper reports on a number of enablers and barriers to access to, and effective participation in, the AHRRTS program.

The findings from this research adds to the current body of evidence on factors involved in the successful implementation of recruitment and retention initiatives for rural and remote allied health. Organisational support and “not having enough time” with all its associated meanings were commonly reported by AHRRTS program stakeholders as key enablers and barriers respectively.

Based on these findings, our recommendations for continued development of rural and remote health workforce training and support programs are:

1. Recognition that time will be a critical barrier requiring targeted enabling strategies. Facilitation of time management and prioritisation through effective engagement with allied health practitioners, line managers, professional supervisors, mentors and peer groups is imperative. Time management and prioritisation training is important as is education around the importance of continuing professional development, particularly with senior clinicians who may not have had prior access to support.
2. In order to remedy organisation support being both an enabler and barrier; our recommendation is twofold: First, engaging with line managers and directors is critical. In particular it is important to ensure that line managers are aware of the program, “on board”, and that initiatives are tailored to line manager priorities in order to facilitate their support of staff participation. Secondly, organisational support can be clearly communicated through existing policies that support participation in continuing professional development including allowances, paid professional development leave [26]. In addition, provision of certificates of attendance and professional contribution to recognise participation is recommended as these are seen as valuable by stakeholders.
3. Finally, to continue to address the persistent barrier associated with travel, and in accordance with the enabler of presentation mode, it is recommended to deliver training via videoconferencing and continue to focus on enhancing the impact of distance based training, including, incorporating strategies to enhance confidence and competence with technology use [27] as this will reduce perceived need to travel and perceived isolation. As presentation mode is of known importance in enabling participation, further emerging forms of delivery (e-learning, blended learning, or other distance-based training modalities which allow participant interaction) should be trialled and evaluated.

## Abbreviations

AHRRTS: Allied Health Rural and Remote Training and Support
AHPEP: Allied Health Professional Enhancement Program
HWA: Health Workforce Australia
SARRAH: Services for Australian Rural and Remote Allied Health.
